# Processing of Paired Click-Tone Stimulation in the Mice Inferior Colliculus

**DOI:** 10.3389/fphys.2019.00195

**Published:** 2019-03-04

**Authors:** Ningqian Wang, Minlin Lin, An Qiao, Zhongju Xiao

**Affiliations:** Key Laboratory of Mental Health of the Ministry of Education, Key Laboratory of Psychiatric Disorders of Guangdong Province, Department of Physiology, School of Basic Medical Sciences, Southern Medical University, Guangzhou, China

**Keywords:** inferior colliculus, paired acoustic stimuli, click, pure tone, post-synaptic current

## Abstract

The inferior colliculus (IC) is known as a neuronal structure involved in the integration of acoustic information in the ascending auditory pathway. However, the processing of paired acoustic stimuli containing different sound types, especially when they are applied closely, in the IC remains poorly studied. We here firstly investigated the IC neuronal response to the paired stimuli comprising click and pure tone with different inter-stimulus (click-tone) intervals using *in vivo* loose-patch recordings in anesthetized BALB/c mice. It was found that the total acoustic evoked spike counts decreased under certain click-tone interval conditions on some neurons with or without click-induced supra-threshold responses. Application of click could enhance the minimum threshold of the neurons responding to the tone in a pair without changing other characteristics of the neuronal tone receptive fields. We further studied the paired acoustic stimuli evoked excitatory/inhibitory inputs, IC neurons received, by holding the membrane potential at -70/0 mV using *in vivo* whole-cell voltage-clamp techniques. The curvature and peak amplitude of the excitatory/inhibitory post-synaptic current (EPSC/IPSC) could be almost unchanged under different inter-stimulus interval conditions. Instead of showing the summation of synaptic inputs, most recorded neurons only had the EPSC/IPSC with the amplitude similar as the bigger one evoked by click or tone in a pair when the inter-stimulus interval was small. We speculated that the IC could inherit the paired click-tone information which had been integrated before reaching it.

## Introduction

In the natural environments, acoustic stimuli are always performed in a temporal complicated context which is affecting their perception and processing in the brain. Paired sounds are commonly adopted to investigate the effect of temporal context on the neuronal responses. Temporal separation of two sounds in a pair could affect the perceptual grouping causing the auditory stream segregation (Bregman, 1990) as well as determine the response to the second sound, i.e., forward masking/suppression (Calford and Semple, 1995; Moore, 1995; Brosch and Schreiner, 1997; Wehr and Zador, 2005). Forward masking has been widely studied in the neuronal auditory pathway including the auditory nerve (Delgutte, 1980), the medial nucleus of the trapezoid body (Gao and Berrebi, 2016), the superior paraolivary nucleus (Gao et al., 2017), the inferior colliculus (IC) (Nelson et al., 2009) and the auditory cortex (Wehr and Zador, 2005). The suppression of neuronal response to the second sound in a pair increases as the temporal sound separation (from the offset of the first sound to the onset of the second one) decreases (Wehr and Zador, 2005; Gao and Berrebi, 2016). However, the neuronal processing and integration of paired acoustic information with different inter-stimulus intervals, especially being given nearly at the same time, are still unclear.

Although forward masking is found in many nuclei (Delgutte, 1980; Wehr and Zador, 2005; Nelson et al., 2009; Gao and Berrebi, 2016; Gao et al., 2017), we focused on the IC in this study considering its integrative function as a synaptic relay station for acoustic information in the neuronal auditory pathway (Malmierca and Hackett, 2010). Paired stimuli comprising of identical sounds are not suitable for identifying the sound processing, especially when they are applied closely, because of the superimposition and/or distortion. Therefore, we here adopted the paired sounds containing a short click and a long pure tone to study the basic features of the IC neuronal responses. The inter-stimulus (click-tone) interval was defined as the time window between the onsets of two sounds in a pair in the present study. Then, no matter how the recorded neurons responded to click, we studied the effect of click on the neuronal tone receptive fields under different inter-stimulus interval conditions.

Identifying the IC sub-threshold response features to the paired sounds will be crucial for understanding the mechanisms underlying the processing and integration of paired acoustic information. Summation of the synaptic inputs evoked by click and tone in a pair with short inter-stimulus intervals should be found on the IC neurons if these acoustic information were integrated on them. To testify this hypothesis, we attempted to investigate the post-synaptic responses induced by paired stimuli using *in vivo* whole-cell recording techniques. Excitatory and inhibitory post-synaptic currents (EPSC and IPSC) were separated by holding the membrane potential at -70 and 0 mV, respectively, in voltage-clamp configuration (Zhou et al., 2010). The peak amplitudes of EPSC/IPSC evoked by click and tone in a pair under different inter-stimulus interval conditions (especially when they were applied nearly at the same time) were analyzed to reveal the mechanisms underlying the processing and integration of paired sounds.

## Materials and Methods

### General

Totally seventy-six female BALB/c mice (aged 4–6 weeks, weighing 16–18 g) without any hearing defects provided by the Experimental Animal Center of Southern Medical University, Guangzhou, China were adopted. Surgery procedures, acoustic stimulation, data acquisition and processing had been approved by the Animal Care and Use Committee of Southern Medical University.

### Surgical Preparation

Atropine sulfate (0.25 mg/kg. Sigma-Aldrich, St. Louis, MO, United States) was injected subcutaneously to reduce tracheal mucous secretion. Fifteen minutes later, urethane (1.2 g/kg. i.p., Sigma-Aldrich, St. Louis, MO, United States) was adopted to anesthetize the animal. The following experiment was conducted on an anti-vibration table placed in a double-walled sound-proof room (air temperature: 24–26°C). We used a stereotaxic apparatus to fix the animal’s head via a 1.5 cm long nail stuck to the dorsal skull surface with dental cement. For the recordings, a 2 × 2 mm area was opened on the skull and the dura was removed under a surgical microscope (WPI, Sarasota, FL, United States) to expose the IC. Vaseline was used to cover the exposed brain during the experiment. The external auditory meatus on the same side of the recording was sealed with dental cement while the pinna on the other side was maintained as in normal animals.

### Acoustic Stimulation

Acoustic stimuli were generated using a TDT 3 (Tucker-Davis Technologies, Alachua, FL, United States) and delivered to the animals via a free-field loudspeaker (ES1, frequency range: 2–110 kHz). The loudspeaker, calibrated by a 1/8 or 1/4 inch microphone (Brüel and Kjaer 4138, 4135, Naerum, Denmark) and an amplifier (Brüel and Kjaer 2610, Naerum, Denmark), was placed 10 cm away from the animal’s head facing the unsealed ear. The frequency, intensity, duration, rise/fall time/function of the acoustic stimuli were controlled manually or automatically via a computer with BrainWare software (Version 9.21. Tucker-Davis Technologies, Alachua, FL, United States).

### *In vivo* Loose-Patch Recording

We adopted *in vivo* loose-patch recordings to investigate the IC neuronal acoustic responses as reported in a previous study (Joshi and Hawken, 2006). Glass micropipettes filled with artificial cerebrospinal fluid (in mM: 124 NaCl, 1.2 NaH_2_PO_4_, 2.5 KCl, 25 NaHCO_3_, 20 glucose, 2 CaCl_2_, 1 MgCl_2_, and 0.5% Biocytin, Sigma-Aldrich, St. Louis, MO, United States. pH 7.2, tip diameter: ∼1 μm, impedance: 6–9 MΩ) were driven by a microdriver (Narishige MO-10, Japan). TDT 3 was used to record, amplify (2000–10000×), filter (band-pass: 0.3–3 kHz) and process the neuronal activities. The shapes and feature spaces (1st to 2nd peak) of spikes (action potentials) were monitored and stored during data acquisition. Neuronal activity was adopted when its signal-noise ratio was greater than 4:1 while the single unit was isolated according to the spike shape similarity.

Broadband noise (frequency: 0–50 kHz; intensity: 90 dB SPL (sound pressure level); duration: 50 ms; rise/fall time: 5 ms; rise/fall function: linear) was firstly applied to detect the neuronal acoustic response. After an IC auditory neuron was found, a frequency-intensity scan was performed by applying pure tones (frequency: 2–64 kHz in 0.1 octave steps; intensity: 10–90 dB SPL in 10 dB steps; duration: 50 ms; rise/fall time: 5 ms; rise/fall function: linear) to the animals (1/s) randomly. The CF (characteristic frequency, the tone frequency at which a neuron responded to the lowest stimulus intensity), MT_tone_ [minimum threshold to tone, the minimum tone intensity at CF eliciting a spike with probability of 0.1 (Keithley and Feldman, 1983)] and BW_10_ (the bandwidth of the tones eliciting response at 10 dB above the MT_tone_) were identified to evaluate the neuronal tone receptive fields. Then, clicks with varying intensities (10–90 dB SPL in 10 dB steps) generated by using 0.1 ms square pulses were delivered randomly (1/s) to test the neuronal response. In this study, a “click +” neuron was termed when it had click-evoked spikes while, otherwise, as a “click -” neuron. On the neurons having click responses, the minimum intensities of clicks eliciting a spike with probability of 0.1 were identified as the MT to click (MT_click_).

To investigate the processing of two sounds on the same neurons, we adopted paired stimuli consisting of a click (80 dB SPL) and a pure tone (at CF, 70 dB SPL, other parameters were same as those used previously in this study). The inter-stimulus intervals were expressed as “delay_click_ - delay_tone_” and the delay_click/tone_ was calculated from the recording onset to the sound onset. We applied the paired sounds with the inter-stimulus intervals ranging from -50 to 100 ms in 10–20 ms steps or in 1–5 ms steps, when they were close, to the animals randomly. Furthermore, a frequency-intensity scan with a click (80 dB SPL) ahead of each tone [inter-stimulus interval as that causing total acoustic evoked spike count (SC_total_) changes by 20%] was performed to reveal the effect of click on the neuronal tone receptive field.

The location of the recorded neuron was confirmed by iontophoretically applied biocytin (1–10 nA for 300 ms ON and 300 ms OFF for 30 min as previous studies; Pinault, 1994, 1996) using microiontophoresis (Neurophore BH-2, Harvard, Holliston, MA, United States). The animal was sacrificed with an overdose of pentobarbital sodium (80 mg/kg, i.p.) and perfused transcardially with saline (0.9%) and fixative (4% paraformaldehyde in 0.1 M PBS, pH 7.4). We removed the brain and fixed it in the same fixative at 4°C overnight, and then for cryoprotection, it was immersed in 20 and 30% sucrose overnight. The brain was cut coronally into 40 μm slices using a freezing microtome (Leica CM 1950, Nussloch, Germany). For staining, the slices were washed by 0.1M PBS (3 × 10 min) and permeabilized with Triton (0.3%) for 2 h to increase permeability of antibody on the cell membrane. After PBS rinsing (3 × 10 min), the slices were incubated with Streptavidin-Cy3 (1:200, Molecular probes, catalog No. 43-4315, United States) for 4 h at room temperature. After being washed and transferred to subbed slides, the sections were stained with 4′,6-diamidino-2-phenylindole (DAPI, 0.25 μg/ml) to visualize the nucleus. Confocal microscopes (Nikon, A1R, Japan) were adopted to examine the location of the recorded neurons. Data from the neurons recorded outside the IC were discarded.

### Whole-Cell Recordings

*In vivo* whole-cell recordings were adopted to reveal the acoustic evoked excitatory/inhibitory synaptic inputs to the IC neurons. Glass microelectrodes containing a cesium-based solution (in mM: 125 Cs-gluconate, 8 phosphocreatine-2Na, 5 TEA-Cl, 4 MgATP, 0.3 GTP, 10 HEPES, 10 EGTA, 1 QX-314, and 2 CsCl) and 0.5% biocytin (pH 7.3, impedance: 4–7 MΩ) were used. By holding the membrane potential at -70 and 0 mV, respectively (Zhou et al., 2010), we separated the excitatory and inhibitory inputs. The IC neuronal activity was recorded for 50 ms before the acoustic stimulation was applied to obtain the baseline. And the baselines obtained from the same neuron for five representations of the identical acoustic stimulation were averaged (expressed as Mean ± SD). EPSC/IPSC was determined when the post-synaptic current changed from the Mean baseline by more than 2 SD.

Paired click-tone stimuli were adopted to explore the relationship between the synaptic inputs induced by different acoustic stimuli. The intensity of click (70 dB) and tone (at CF, 80 dB) in each paired stimulation were fixed while their intervals (delay_click_ – delay_tone_) changed from -100 to 100 ms in 10–20 ms steps or in 1–5 ms steps when they were close. The recorded neurons were labeled with biocytin and data from neurons within the IC were further analyzed.

### Data Processing

The waveforms of the neuronal spikes were collected and stored as data sets. The IC neuronal responses recorded extracellularly to five presentations of identical acoustic stimulation (including paired stimuli) were displayed as post-stimulus time histograms (PSTH). The acoustic evoked SCs were calculated within a 100 ms time window started at the stimulus onset (Liang et al., 2011).

To investigate the processing of click and tone in a pair on the same neurons, we plotted the SC_total_ as a function of inter-stimulus intervals and compared the SC_total_s evoked by paired sounds with three inter-stimulus intervals (-40, 1 and 40 ms). Then, the CF, MT and BW_10_ of the IC neuronal tone receptive field with and without a click before each tone with a certain inter-stimulus interval (causing SC_total_ changes by 20%) were compared to evaluate the effects of clicks on the neuronal receptive fields to tones. Finally, we analyzed the relationship between excitatory/inhibitory inputs (EPSCs/IPSCs) evoked by paired click-tone stimulations with different inter-stimulus intervals.

We used Excel 2007 and OriginPro 7.5 to calculate the values of relevant parameters and data fitting and plotting. One-way ANOVA was used to compare means and LSD’s test was used for multiple comparisons. Paired-samples T Test was also adopted to compare means of paired samples in this study. *P* < 0.05 was concerned as significant difference.

## Results

### IC Neuronal Responses to Clicks and/or Pure Tones

**FIGURE 1:**
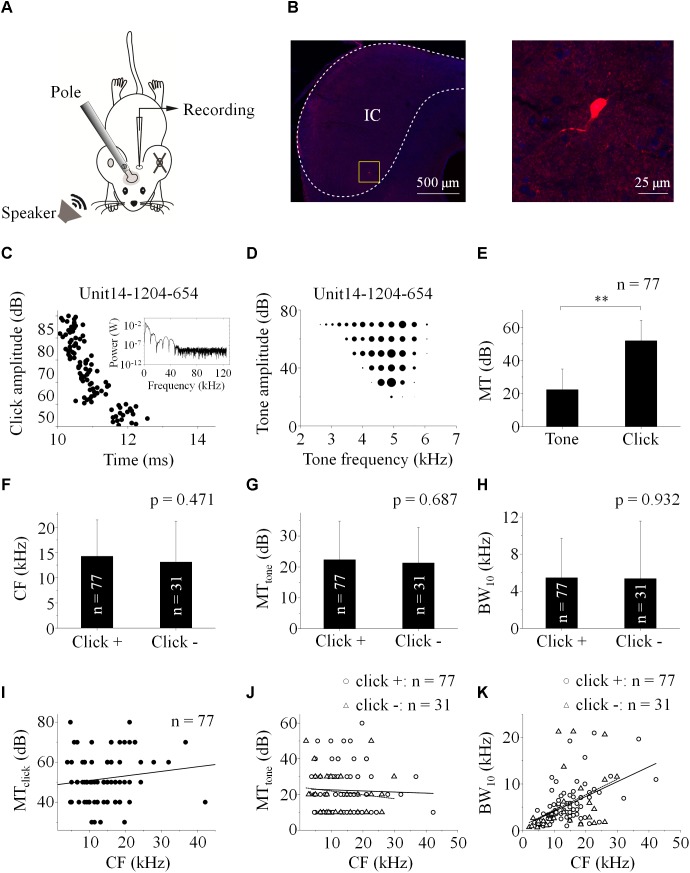
Basic features of extracellularly recorded IC neuronal acoustic response. **(A)** Experimental setup. *Pole*: fixing animal’s head. **(B)** A biocytin-labeled neuron in the IC (*Left*, Scale bar: 500 μm. Indicated by the rectangle. *Right*: Enlarged figure, Scale bar: 25 μm). Neuronal responses (e.g., Unit14-1204-654) were induced by clicks [raster plotting, **(C)** *Inserted*: fast Fourier transform of the click adopted in this study] and pure tones (receptive field, **D**). **(E)** The MTs of the neurons responding to clicks (51.82 ± 12.22 dB SPL) were higher than those to tones (22.34 ± 12.45 dB SPL. One-way ANOVA, p < 0.001). **(F–H)** Basic features of the neuronal tone receptive fields. Distributions of CF **(F)**, MT_tone_ **(G)** and BW_10_ **(H)** of the neurons with (“click +”: *n* = 77) and without (“click -”: *n* = 31) click-evoked spikes were similar (14.25 ± 7.22 and 13.10 ± 8.09 kHz, 22.34 ± 12.45 and 21.29 ± 11.47 dB SPL, 5.46 ± 4.25 and 5.37 ± 6.20 kHz, One-way ANOVA, *p* = 0.471, 0.687 and 0.932, respectively). **(I)** On the “click +” neurons, the MTs to clicks were plotted as a function of their CFs responding to tones (linear fit, *line*, *n* = 77, *R*^2^ = 0.005, *P* = 0.243). **(J)** The MTs to tones on the IC neurons had no relationship with their CFs (linear fit, total: *n* = 108, *R*^2^ = -0.005, *p* = 0.498. In detail, “click +” neurons: *line*, *n* = 77, *R*^2^ = -0.012, *p* = 0.760; “click -” neurons: *dashed line*, *n* = 31, *R*^2^ = -0.011, *p* = 0.415). **(K)** The BW_10_ increased along with the CF (linear fit, total: *n* = 108, *R*^2^ = 0.240, *p* < 0.001. In detail, “click +”: *line*, *n* = 77, *R*^2^ = 0.286; *p* < 0.001; “click -”: *dashed line*, *n* = 31, *R*^2^ = 0.162; *p* = 0.014). ^∗∗^*p* < 0.01.

Totally one hundred and eight IC neurons recorded in anesthetized mice (Figure 1A) had responses to the pure tones in this study and showed as disk-shaped cells (Oliver et al., 1991) sampled as in Figure 1B. The recording depth ranged from 232 to 1580 μm beneath the brain surface. The CF, MT_tone_ and BW_10_ were 2 – 42.22 (13.92 ± 7.46) kHz, 10 – 60 (22.04 ± 12.13) dB SPL and 0.59 – 21.30 (5.43 ± 4.86) kHz, respectively.

Among these neurons, 77 (71.30%) also had spikes evoked by clicks (“click +” neurons., e.g., Unit14-1204-654, click response: Figure 1C; tone response: Figure 1D). The MTs to clicks (MT_click_, 51.82 ± 12.22 dB SPL) were higher than those to tones (MT_tone_, 22.34 ± 12.45 dB SPL) on them (One-way ANOVA, *p* < 0.001, Figure 1E). Between the neurons with (“click +”: *n* = 77) and without (“click -”: *n* = 31) click-evoked spikes, no significant differences were found in CFs (14.25 ± 7.22 and 13.10 ± 8.09 kHz, One-way ANOVA, *p* = 0.471, Figure 1F), MT_tone_s (22.34 ± 12.45 and 21.29 ± 11.47 dB SPL, One-way ANOVA, p = 0.687, Figure 1G) and BW_10_s (5.46 ± 4.25 and 5.37 ± 6.20 kHz. One-way ANOVA, *p* = 0.932, Figure 1H). Neurons with different CFs had similar MTs to clicks (linear fit: *R*^2^ = 0.005, *p* = 0.243, Figure 1I) or tones [linear fit: total: *R*^2^ = -0.005, *p* = 0.498 (*not shown*). “click +” neurons: *R*^2^ = -0.012, *p* = 0.760 (*line*); “click -” neurons: *R*^2^ = -0.011, *p* = 0.415 (*dashed line*), Figure 1J]. However, as the CFs of the recorded neurons increased, the neurons with or without click-induced spikes had more broad-tuned receptive fields by showing increasing BW_10_ [linear fit: total: *R*^2^ = 0.240, *p* < 0.001 (*not shown*). “click +” neurons: *R*^2^ = 0.286, *p* < 0.001 (*line*); “click -” neurons: *R*^2^ = 0.162, *p* = 0.014 (*dashed line*), Figure 1K].

### IC Neuronal Responses to Paired Click-Tone Stimulation

#### Effect of the Inter-Stimulus Interval of Paired Stimuli on the IC Neuronal Response

In the present study, we adopted paired stimuli comprising a click (80 dB SPL) and a tone (at CF, 70 dB SPL) with different intervals (delay_click_ – delay_tone_) to identify the basic features of the processing of them on the IC neurons.

On the IC neurons showing spikes evoked by both click and tone in a pair (“click +” neurons: *n* = 77, e.g., Figure 1C,D), it is hard to separate the click-evoked spikes from those induced by tones when the inter-stimulus intervals were small. Therefore, on them, we calculated the total SC (SC_total_) evoked by paired click and tone. The SC_total_ obtained when the click was 50 ms earlier than the tone in a pair was adopted to normalize those under other inter-stimulus interval conditions by dividing them. In this study, the significant changes of neuronal responses were considered as that the evoked SC_total_ varied by more than 20% of the reference.

**FIGURE 2:**
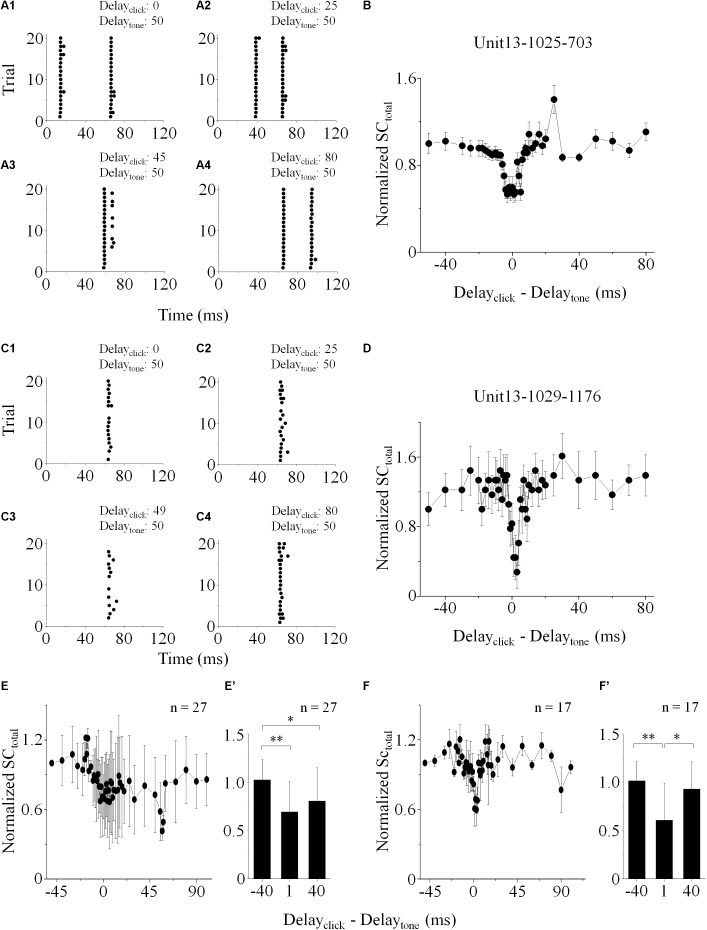
IC neuronal responses to paired stimuli with different inter-stimulus intervals. Acoustic responses on the “click +” (e.g., Unit13-1025-703, **A,B**) and “click -” (e.g., Unit13-1029-1176, **C,D**) neurons were shown. **(A,C)** Raster plotting of responses to paired sounds with different inter-stimulus intervals (delay_click_ – delay_tone_: -50, -25, -5/-1, and 30 ms). The SC_total_ (total SC induced by paired sounds), measured when click was 50 ms before tone in a pair, was adopted to normalize those under other inter-stimulus interval conditions. On the neurons with (e.g., Unit13-1025-703, **B**; total: *n* = 27, **E**) or without (e.g., Unit13-1029-1176, **D**; total: *n* = 17, **F**) click evoked spikes, the normalized SC_total_ decreased when the click and tone in a pair were close. The normalized SC_total_ obtained as the inter-stimulus intervals were -40, 1, and 40 ms were compared on the “click +” (*n* = 27, **E’**. One-way ANOVA, *p* = 0.011; LSD’s test for multiple comparison, -40_1: *p* = 0.007; -40_40: *p* = 0.017; 1_40: *p* = 0.337) and “click -” neurons (*n* = 17, **F’**. One-way ANOVA, *p* = 0.013; LSD’s test, -40_1: *p* = 0.004; -40_40: *p* = 0.382; 1_40: *p* = 0.018). ^∗^*p* < 0.05, ^∗∗^*p* < 0.01.

The normalized SC_total_ decreased when the paired sounds were given to the animal with short inter-stimulus intervals on 35.06% of the recorded “click +” neurons (*n* = 27, Figure 2A,B,E). On these neurons, the normalized SC_total_ evoked by paired sounds with -40, 1, and 40 ms click-tone intervals were compared (Figure 2E’. One-way ANOVA, *p* = 0.011; LSD’s test, -40_1: *p* = 0.007; -40_40: *p* = 0.017 and 1_40: *p* = 0.337). It indicated that the processing of click and tone in a pair on these IC neurons had interactions when they were close. No significant difference was found in the CFs of the “click +” neurons with (*n* = 27, 13.25 ± 7.15 kHz) and without (*n* = 50, 14.79 ± 7.27 kHz) the interactions between the click and tone responses (One-way ANOVA, *p* = 0.375).

We used the same procedure to test if it is also the case on the neurons without any spikes induced by clicks (“click -” neurons: *n* = 31). Since these neurons only had tone-evoked spikes, SC_total_ equaled SC_tone_. The SC_total_ decreased significantly when paired sounds had small click-tone intervals on seventeen “click -” neurons (54.84%, Figure 2C,D,F. Comparison of normalized SC_total_ with -40, 1 and 40 ms inter-stimulus intervals: One-way ANOVA, *p* = 0.013; LSD’s test, -40_1: *p* = 0.004; -40_40: *p* = 0.382 and 1_40: *p* = 0.018, Figure 2F’). These results suggested that click-evoked sub-threshold inputs should affect the responses of the “click -” neurons to tones. Similar as the “click +” neurons, there was no significant difference in the CFs between the “click -” neurons with (*n* = 17, 14.00 ± 7.39 kHz) and without (*n* = 14, 12.00 ± 9.14 kHz) the effects of clicks on neuronal tone responses (One-way ANOVA, *p* = 0.504).

The power spectrum of the click used in this study was majorly less than 10 kHz (*inserted* in Figure 1C). So, “10 kHz” was adopted as a cutoff to categorize the recorded IC neurons based on their receptive frequency ranges at 80 dB SPL (Table 1). The “click +/-” neurons (*n* = 77/31) with the receptive ranges higher than, lower than or cross 10 kHz were categorized, and so did the neurons showing interactions between the processing of the paired click-tone stimulation (*n* = 27/17). We found that, even on the neurons having receptive ranges higher than 10 kHz, the processing of click and tone in a pair had interactions (“click +” neurons: 6 out of 23; “click -” neurons: 4 out of 6). It indicated that the clicks might widely provide synaptic inputs to the IC neurons, even to those with receptive fields located apart from the major click frequency range.

**Table 1:** Features of frequency ranges of the neuronal tone receptive field at 80 dB SPL.

	**Cell number**		
	**Click +**	**Click -**	**Summary**
<10 kHz	15(8)	14(7)	29(15)
>10 kHz	23(6)	6(4)	29(10)
Cross 10 kHz	39(13)	11(6)	50(19)
	77(27)	31(17)	108(44)


*Tone responsive neurons recorded extracellularly with (*n* = 77) or without (*n* = 31) click-evoked spikes were categorized based on their frequency receptive fields at 80 dB SPL. Three categories: more than, less than, and cross 10 kHz. Neurons having interactions between the processing of click and tone (*n* = 44) were also categorized (cell numbers were shown in *brackets*)*.

#### Effects of Click on the IC Neuronal Receptive Field to Pure Tone

To reveal the mechanism underlying the affection of the click-tone interval on the neuronal response, we investigated the effect of click on the IC neuronal receptive field to pure tone by performing two frequency-intensity scans with or without a given click (80 dB SPL) before each tone. The inter-stimulus intervals were set as those causing the acoustic evoked SC_total_ changes by 20% in the decreasing phase. The CF, MT_tone_ and BW_10_ were adopted to evaluate the location and shape of neuronal tone receptive fields in the coordinate system.

**FIGURE 3:**
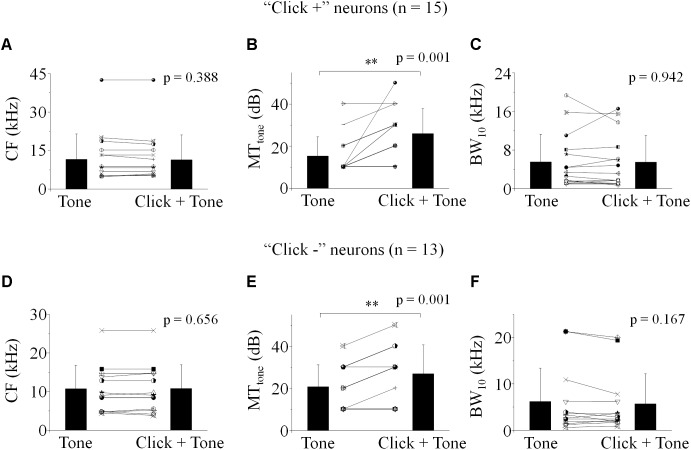
Effect of click on the basic feature of IC neuronal tone receptive field. **(A–C)** The “click +” neurons (*n* = 15). **(D–F)** The “click -” neurons (*n* = 13). CF **(A,D)**, MT_tone_ **(B,E)** and BW_10_ **(C,F)** obtained with and without click ahead of pure tone were compared. No differences were found in the CFs **(A,D)** and BW_10_s **(C,F)** before and after click was applied before each tone [Paired-samples *T*-Test (two-tailed), CF: “click +”: *p* = 0.388; “click -”: *p* = 0.656. BW_10_: “click +”: *p* = 0.942; “click -”: *p* = 0.167]. However, the MT_tone_ of neuronal responses to tones was significantly increased by the application of click [**B,E**, Paired-samples *T*-Test (two-tailed), “click +”: *p* = 0.001; “click -”: *p* = 0.001]. ^∗∗^*p* < 0.01.

Both CF and BW_10_ were not affected by the click application on some neurons with [*n* = 15, Figure 3A,C. Paired-samples *T*-test (two-tailed), *p* = 0.388, 0.942] or without [*n* = 13, Figure 3D,F, Paired-samples *T*-test (two-tailed), *p* = 0.656, 0.167] click evoked supra-threshold responses. It indicated that the clicks could have no effects on the horizontal location and shape of the IC neuronal tone receptive fields in the coordinate system. Interestingly, clicks could shift the tone receptive fields upward vertically by enhancing the MT_tone_ on these “click +” [*n* = 15, Figure 3B, Paired-samples *T*-test (two-tailed), *p* = 0.001] or “click -” neurons [*n* = 13, Figure 3E, Paired-samples *T*-test (two-tailed), *p* = 0.001]. To sum up, the neuronal responses to the tones with different frequencies could be widely inhibited by the application of clicks by showing corresponding increased MTs. However, the corresponding synaptic mechanisms underlying this inhibition is still unclear.

### Synaptic Inputs Evoked by Click and Tone in a Pair to the IC Neurons

We recorded the synaptic inputs evoked by paired acoustic stimuli to the IC neurons by using *in vivo* whole-cell recording techniques (in the voltage-clamp configuration). The excitatory and inhibitory post-synaptic currents were isolated by holding the membrane potential at -70 and 0 mV, respectively (Wu et al., 2008). In this study, we totally recorded the paired click- and/or tone-induced PSCs on eleven IC neurons (CF: 16.64 ± 8.22 kHz). And only on six of them (CF: 15.61 ± 4.30 kHz), both acoustic evoked EPSCs and IPSCs were recorded. We attempted to reveal the relationship between the EPSCs/IPSCs evoked by the sounds in a pair with different intervals on these six neurons.

**FIGURE 4:**
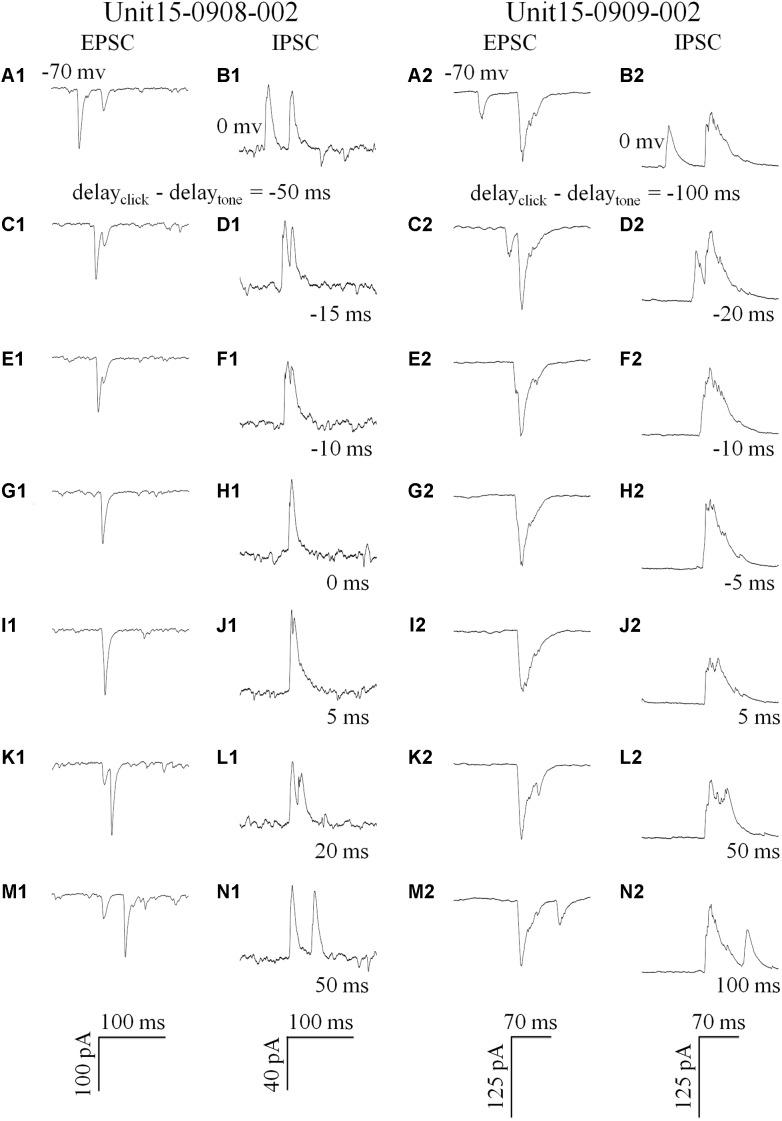
Synaptic inputs induced by paired stimulation with different inter-stimulus intervals (Examples: 1: Unit15-0908-002 and 2: Unit15-0909-002). Five repeats of EPSCs (holding at -70 mv, **A,C,E,G,I,K,M**) and IPSCs (holding at 0 mv, **B,D,F,H,J,N**) were averaged. Click and tone evoked EPSCs **A1** and IPSCs **(B1/2,N1/2)** were apart from each other when the inter-stimulus intervals (delay_click_ – delay_tone_) were large (Unit15-0908-002: -50 ms, **A1/B1**, 50 ms, **M1/N1**; Unit15-0909-002: -100 ms, A2/B2, 100 ms, **M2/N2**). And they were partially overlapped when the click was moved towards **(C1/2–F1/2)** or away from the tone in a pair **(G1/2–L1/2)**. When the click and tone in a pair were applied to the animal with a short interval or at the same time (0 ms: **G1/H1** and -5 ms: **G2/H2**), the PSCs evoked by tone or click were completely overlapped by showing only one PSC.

**FIGURE 5:**
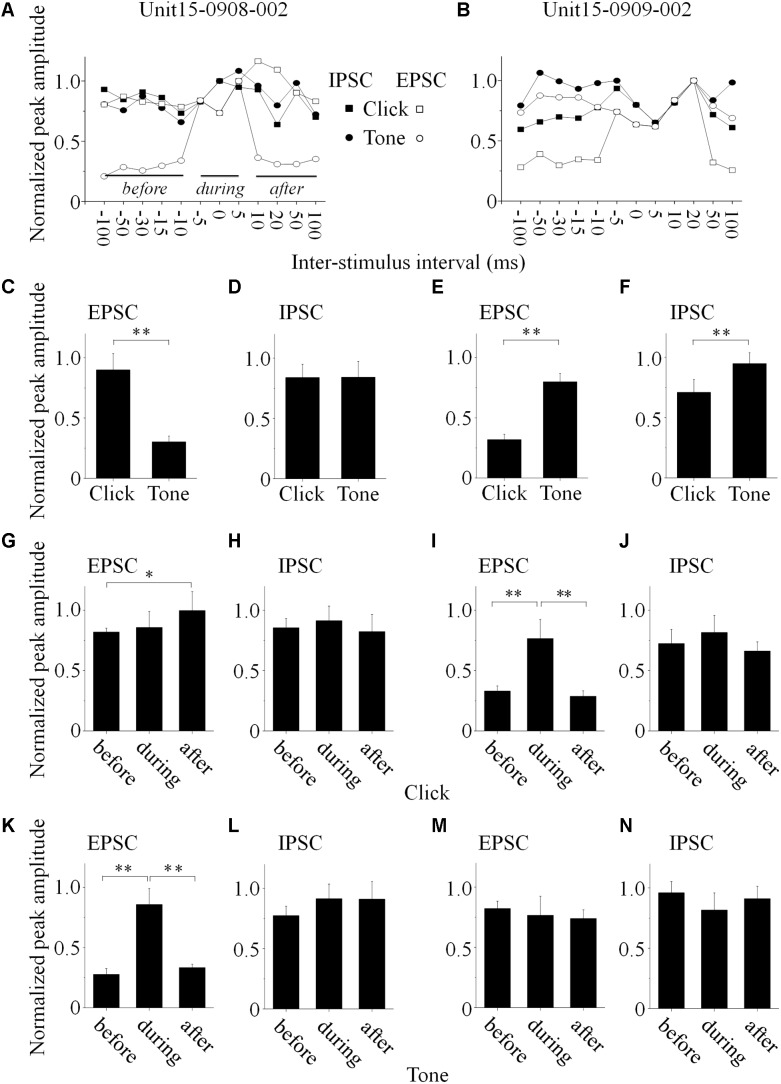
Basic features of the synaptic inputs evoked by paired acoustic stimuli (data obtained from the same neurons as in Figure 4, Unit15-0908-002: **A,C,D,G, H,K,L**; Unit15-0909-002: **B,E,F,I,J,M,N**). **(A,B)** The maximal PSC peak amplitude, obtained when the click and tone evoked PSCs were completely overlapped, was set as a reference to normalize all the PSCs evoked by paired sounds under different inter-stimulus intervals (*solid lines* indicated the periods before, during and after the completely overlapping). On the Unit15-0908-002, click evoked EPSCs bigger than those by tone (**C**, One-way ANOVA, *p* < 0.001) while IPSCs evoked by paired sounds had similar peak amplitudes (**D**, One-way ANOVA, *p* = 0.967). Meanwhile, tones evoked EPSCs (**E**, One-way ANOVA, *p* < 0.001) and IPSCs (**F**, One-way ANOVA, *p* < 0.001) bigger than those by clicks on the Unit15-0909-002. The peak amplitudes of paired click **(G–J)** and tone evoked PSCs **(K–N)** before, during and after the completely overlapping with those induced by tone or click were compared (One-way ANOVA, and LSD’s test was used for multiple comparison). Clicks evoked PSCs with peak amplitudes similar as the completely overlapped ones on the Unit15-0908-002 [EPSCs: **(G)** One-way ANOVA, *p* = 0.104; LSD’s test, before_during: *p* = 0.653; during_after: *p* = 0.137; before_after: *p* = 0.042; IPSCs: **(H)** One-way ANOVA, *p* = 0.685; LSD’s test, before_during: *p* = 0.563; during_after: *p* = 0.376; before_after: *p* = 0.672]. Meanwhile, on the same neuron, tones evoked smaller EPSCs (**K**, One-way ANOVA, *p* < 0.001; LSD’s test, before_during: *p* < 0.001; during_after: *p* < 0.001; before_after: *p* = 0.276) and similar IPSCs (**L**, One-way ANOVA, *p* = 0.195; LSD’s test, before_during: *p* = 0.190; during_after: *p* = 0.968; before_after: *p* = 0.102) comparing with the completely overlapped ones. However, on the Unit15-0909-002, clicks evoked smaller EPSCs (**I**, One-way ANOVA, *p* < 0.001; LSD’s test, before_during: *p* < 0.001; during_after: *p* = 0.001; before_after: *p* = 0.655) and similar IPSCs (**J**, One-way ANOVA, *p* = 0.347; LSD’s test, before_during: *p* = 0.280; during_after: *p* = 0.185; before_after: *p* = 0.553) comparing to the completely overlapped PSCs. And tones evoked PSCs with similar peak amplitudes under all the inter-stimulus interval conditions [EPSCs: **(M)** One-way ANOVA, *p* = 0.634; LSD’s test, before_during: *p* = 0.463; during_after: *p* = 0.790; before_after: *p* = 0.415; IPSCs: **(N)** One-way ANOVA, *p* = 0.156; LSD’s test, before_during: *p* = 0.079; during_after: *p* = 0.360; before_after: *p* = 0.603]. ^∗^*p* < 0.05, ^∗∗^*p* < 0.01.

**FIGURE 6:**
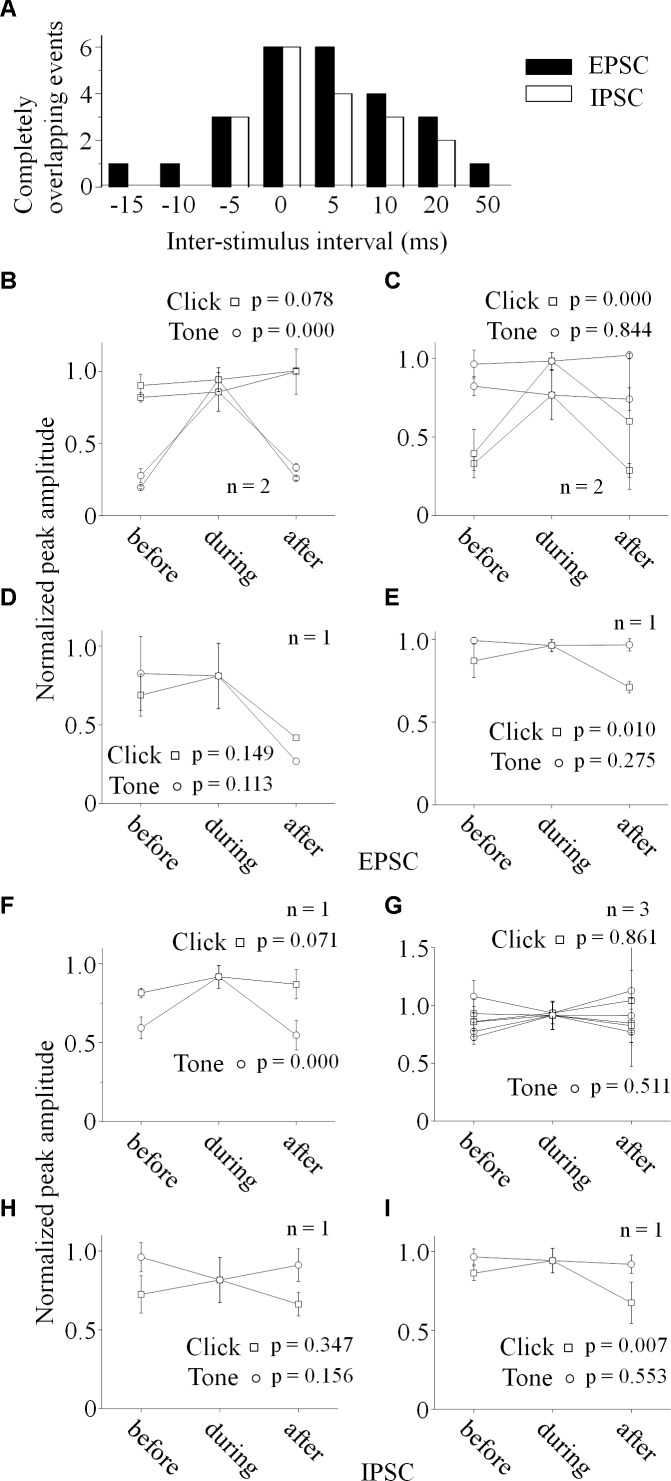
Relationship between the paired stimuli evoked synaptic inputs. **(A)** Distributions of the inter-stimulus intervals of the paired sounds for the completely overlapping of the evoked PSCs (*Filled*: EPSC, *Empty*: IPSC). Relationships between the paired click-tone evoked EPSCs **(B–E)** and IPSCs **(F–I)** were analyzed by comparing the PSC peak amplitudes before, during and after the completely overlapping on the six neurons with both recorded EPSCs and IPSCs. The peak amplitudes of the completely overlapped EPSCs were similar as the relative large EPSCs evoked by click (**B**, *n* = 2, One-way ANOVA, click: *p* = 0.078, tone: *p* < 0.001) or tone (**C**, *n* = 2, One-way ANOVA, click: *p* < 0.001, tone: *p* = 0.844). click and tone evoked EPSCs, the completely overlapped EPSC amplitudes were similar as those evoked by click and tone (**D**, One-way ANOVA, click: *p* = 0.149, tone: *p* = 0.113). **(E)** Although the completely overlapped EPSC amplitudes were similar as the EPSCs evoked clicks rather than tones (*n* = 1, One-way ANOVA, click: *p* = 0.010, tone: *p* = 0.275), the tone evoked EPSC amplitudes changed as a function of inter-stimulus interval in a complicated way (LSD’s test, before_during: *p* = 0.130; during_after: *p* = 0.004; before_after: *p* = 0.019). **(F)** The completely overlapped IPSC amplitudes were similar as the larger ones evoked by clicks (*n* = 1, One-way ANOVA, click: *p* = 0.071, tone: *p* < 0.001). **(G)** On the neurons with similar IPSCs induced by the paired stimuli (*n* = 3), all the IPSCs obtained under different inter-stimulus intervals had no significant differences in the peak amplitudes (One-way ANOVA, click: *p* = 0.961, tone: *p* = 0.511). **(H)** The completely overlapped IPSC amplitudes fell in between those induced by click and tone (One-way ANOVA, click: *p* = 0.347, tone: *p* = 0.156) on the neuron with larger tone responses (the same neuron as in Figure 5J,N). **(I)** Although the completely overlapped IPSC amplitudes were similar as the larger ones evoked by tones (One-way ANOVA, click: *p* = 0.007, tone: *p* = 0.553), the inter-stimulus intervals had complicated effects on the tone-evoked IPSCs (LSD’s test, before_during: *p* = 0.189; during_after: *p* = 0.003; before_after: *p* = 0.009).

Click and tone evoked PSCs (EPSC, Figure 4A1/2; IPSC, Figure 4B1/2) were totally apart from each other when the click was applied to the animal 50 (e.g., Unit15-0908-002, Figure 4A1,B1) or 100 ms (e.g., Unit15-0909-002, Figure 4A2,B2) earlier than the tone in a pair. The PSCs evoked by paired sounds started to be partially overlapped with each other as the inter-stimulus intervals decreased (Figure 4C–F). And interestingly, they were completely overlapped by showing only one PSC when the paired sounds were given with relative small inter-stimulus intervals (e.g., Unit15-0908-002: 0 ms, Figure 4G1/H1; Unit15-0909-002: -5 ms, Figure 4G2/H2). The click-tone intervals of paired stimuli causing the completely overlapping of PSCs on the same neurons could be different (e.g., Unit15-0908-002, EPSC: -5, 0, 5 ms, IPSC: -5, 0 ms, Figure 5A; Unit15-0909-002, EPSC: -5, 0, 5, 10, 20 ms, IPSC: 0, 5, 10, 20 ms, Figure 5B) and majorly between -5 and 10 ms on the six neurons for further analysis (Figure 6A). The disappeared PSCs seemed have lower amplitudes (Figure 4A–H) and reappeared when the click was moved away from the tone in a pair (Figure 4I–N).

PSC peak amplitudes for five presentations of identical acoustic stimulation were averaged and the maximal amplitude of completely overlapped PSC was set as a reference to normalize the PSCs obtained under different click-tone intervals by dividing them with it. The normalized peak amplitudes were plotted as a function of the inter-stimulus interval of the paired sounds (Figure 5A,B, the same neurons as in Figure 4). On the same neurons, the relationship between the peak amplitudes of EPSC and IPSC could be consistent (*n* = 3. e.g., Unit15-0909-002, the tone-evoked PSCs were higher than those induced by clicks, One-way ANOVA, *p* < 0.001 for both EPSC and IPSC, Figure 4A2,B2, 5E,F), or not (*n* = 3., e.g., Unit15-0908-002, click evoked higher EPSC and similar IPSC as tone, One-way ANOVA, *p* < 0.001 and *p* = 0.967, respectively, Figure 4A1,B1, 5C,D).

To reveal the relationship between PSCs evoked by paired stimuli, we compared the peak amplitudes of PSCs induced by click (Figure 5G–J, 6B–I) or tone (Figure 5K–N, 6B–I) in a pair before, during and after they were completely overlapped (e.g., Unit15-0908-002, EPSC: indicated by *solid lines* in Figure 5A. One-way ANOVA and LSD’s test were used). On those neurons that click and tone in a pair evoked excitatory inputs with different amplitudes (*n* = 5, Figure 6B,C,E), the relative bigger EPSCs evoked by click (*n* = 2, Figure 6B; One-way ANOVA, *p* = 0.078; e.g., Unit15-0908-002, Figure 5G; One-way ANOVA, *p* = 0.104) or tone (*n* = 2, Figure 6C; One-way ANOVA, p = 0.844; e.g., Unit15-0909-002, Figure 5M; One-way ANOVA, *p* = 0.634) were similar under all click-tone interval conditions. The corresponding smaller EPSCs induced by tone (Figure 6B; One-way ANOVA, *p* < 0.001; e.g., Unit15-0908-002, Figure 5K; One-way ANOVA, *p* < 0.001; LSD’s test, before_during: *p* < 0.001; during_after: *p* < 0.001; before_after: *p* = 0.276) or click (Figure 6C; One-way ANOVA, *p* < 0.001; e.g., Unit15-0909-002, Figure 5I; One-way ANOVA, *p* < 0.001; LSD’s test, before_during: *p* < 0.001; during_after: *p* = 0.001; before_after: *p* = 0.655) disappeared, instead of being superimposed with the higher ones, when the inter-stimulus intervals were small. Even on the neuron (Unit15-0907-001, Figure 6E) having complex EPSC amplitude changes along with the inter-stimulus intervals, no synaptic input summations were found (click evoked EPSCs: One-way ANOVA, *p* = 0.010; LSD’s test, before_during: *p* = 0.130; during_after: *p* = 0.004; before_after: *p* = 0.019; tone evoked EPSCs: One-way ANOVA, *p* = 0.275). Meanwhile, the click and tone evoked EPSCs with similar amplitudes (Unit15-0909-001, Figure 6D) were also not superimposed with each other (click evoked EPSCs: One-way ANOVA, *p* = 0.149; tone evoked EPSCs: One-way ANOVA, *p* = 0.113) when the paired sounds were applied closely.

The situations for IPSC (Figure 6F–I) were similar as those for EPSC (Figure 6B–E) but not necessarily on the same neurons. Among the neurons having different amplitudes of inhibitory inputs evoked by click and tone (*n* = 3), one had almost unchanged click induced IPSCs (Unit15-0909-001, Figure 6F; One-way ANOVA, *p* = 0.071) under different inter-stimulus interval conditions. And even on the other two neurons (Unit15-0909-002, Figure 5F,J,N, 6H and Unit15-0910-001, Figure 6I) with complex IPSC amplitude changes, there were no input summation when the click and tone in a pair were applied closely. On one of them (Unit15-0909-002, Figure 5J,N, 6H), the peak amplitudes of completely overlapped IPSCs fell in between those induced by click (Figure 5J, 6H, One-way ANOVA, *p* = 0.347) and tone (Figure 5N, 6H, One-way ANOVA, *p* = 0.156) while more complicated changes were shown on the other one (Unit15-0910-001, Figure 6I, click evoked IPSCs: One-way ANOVA, *p* = 0.007; LSD’s test, before_during: *p* = 0.189; during_after: *p* = 0.003; before_after: *p* = 0.009; tone evoked IPSCs: One-way ANOVA, *p* = 0.553). On the neurons who had similar amplitudes of click and tone evoked IPSCs (*n* = 3, Figure 6G, e.g., Unit15-0908-002, Figure 5D. One-way ANOVA, *p* = 0.967), the peak amplitudes of the completely overlapped IPSCs had no difference with the incompletely overlapped ones evoked by click (Figure 6G, One-way ANOVA, *p* = 0.961; e.g., Figure 5H, One-way ANOVA, *p* = 0.685) and tone (One-way ANOVA, *p* = 0.511; e.g., Figure 5L, One-way ANOVA, *p* = 0.195).

These results suggested that some IC neurons only responded to a single acoustic stimulus (click or tone) in a pair which induced the relative larger synaptic inputs when the paired sounds were applied nearly at the same time.

## Discussion

### Properties of Neuronal Click Perceptions in the IC

Tonotopic organization and sharp frequency tuning in the IC have been widely studied in the cat (Aitkin et al., 1975; Semple and Aitkin, 1979; Serviere and Webster, 1981; Brown et al., 1997; Schreiner and Langner, 1997), gerbil (Ryan et al., 1982; Grana et al., 2017), monkey (FitzPatrick, 1975), bat (Zook et al., 1985; Poon et al., 1990), rat (Clopton and Winfield, 1973; Huang and Fex, 1986; Malmierca et al., 2008), mouse (Stiebler and Ehret, 1985), and human (De Martino et al., 2013; Ress and Chandrasekaran, 2013). As a powerful acoustic stimulation, click could generally evoked the responses on the IC neurons whose receptive frequency range at 80 dB had intersections with the click power spectrum (majorly < 10 kHz, Figure 1 and Table 1). Interestingly, even on those neurons whose receptive frequency ranges (at 80 dB) were away from the click power spectrum, clicks could also provide synaptic inputs (Table 1) by showing supra-threshold spikes (“click +” neuron, Figure 1) or sub-threshold inputs affecting the neuronal tone response (“click -” neuron, Figure 1, 2C,D,F). We therefore speculate that, in addition to the tonotopic organized lemniscal pathway, the click information might reach the IC via the non-lemniscal pathway.

### Properties of Click Affecting the IC Neuronal Response to the Tone in a Pair

We found in this study that even on those IC neurons only having the supra-threshold responses to tones (i.e., “click -” neurons), the application of click could depress their tone responses (Figure 2C,D,F,F’). And these effects were depending on the inter-stimulus intervals, several to tens of milliseconds, between the click and tone in a pair no matter which sound was applied early (Figure 2F,F’). It should be the sub-threshold response evoked by click causing these changes of the IC tone response by enhancing their MT_tone_s On a neuron with similar (Figure 3D–F).

Although the SCs evoked by click and tone in a pair were adopted to evaluate the interactions between them on the “click +” neurons, the changes of SC_total_ were similar as those on the “click -” neurons (Figure 2). The clicks enhanced the MT_tone_ without changing the CF and BW_10_ of the neuronal tone receptive field (Figure 3A–C). Therefore, we speculated that the mechanisms underlying the effects of clicks on the tone response on the neurons with and without click evoked supra-threshold spikes should be similar. Since the spikes evoked by click and tone were impossible to be separated when they were closely on the “click +” neurons, we did not compare the inter-stimulus intervals of the paired stimuli for suppression between the neurons with and without click-evoked spikes in this study.

### Mechanism Underlying the Processing of Paired Click-Tone Stimulation on the IC Neurons

Although previous studies had indicated that a delayed, long-lasting GABAergic inhibition should be involved in the forward masking formation in the sensory cortex (Swadlow, 2003; Wehr and Zador, 2005; Isaacson and Scanziani, 2011), the integrative patterns of the excitatory synaptic inputs induced by paired sounds remains unclear, and neither does the inhibitory inputs. In this study, by holding membrane potentials at different levels (-70 and 0 mV, respectively), we separated the acoustic (click/tone) evoked EPSC and IPSC focusing on their peak amplitude changes along with the inter-stimulus intervals of paired sounds.

If the integration of paired acoustic information with relative short inter-stimulus intervals takes place in the IC, the summation of the sub-threshold responses (EPSC or IPSC) should occur on the recorded IC neurons by showing the changes in the curvature and peak amplitude as a function of the inter-stimulus intervals. In another word, the PSCs induced by the two sounds in a pair should be superimposed with each other when the inter-stimulus intervals are short. However, in spite of the small sample size (*n* = 6, Figure 6), our present *in vivo* whole-cell recording results indicate that it is not the case on most recorded IC neurons by showing relative unchanged EPSC (*n* = 5, Figure 4, 5G,K,I,M, 6B–D) or IPSC (*n* = 4, Figure 4, 5H,L, 6F,G) under different click-tone interval conditions. Although the paired click and tone could evoke PSCs with different peak amplitudes, when the paired sounds are given closely, the bigger EPSC/IPSC induced by click or tone completely overlapped the relative small one without obvious superimposition (Figure 4, 5G–I,K–M, 6B–D,F,G). Only a few exceptions for the complicated changes in EPSC (*n* = 1, Figure 6E) or IPSC (*n* = 2, Figure 6H,I) evoked by paired sounds with different click-tone intervals were found. Therefore, we have reasons to believe that, at least, part of the whole-cell recorded neurons in this study inherit the paired acoustic information, especially when they are relatively close to each other, integrated before it reaches the IC. Basilar membrane and auditory nuclei in the ascending auditory pathway below the IC might be the candidates for the acoustic information integration which needs further investigations.

## Data Availability

The datasets generated and/or analyzed during the current study are available from the corresponding author on reasonable request.

## Author Contributions

ZX conceived and designed the study. ML and AQ performed the experiments. NW and ML analyzed the data. NW wrote the manuscript. ZX reviewed and edited the manuscript. All authors read and approved the manuscript.

## Conflict of Interest Statement

The authors declare that the research was conducted in the absence of any commercial or financial relationships that could be construed as a potential conflict of interest.
